# The effect of special educational assistance in early childhood education and care on psycho-social difficulties in elementary school children

**DOI:** 10.1186/s13034-022-00442-5

**Published:** 2022-02-24

**Authors:** Guido Biele, Ratib Lekhal, Kristin R. Overgaard, Mari Vaage Wang, Ragnhild Eek Brandlistuen, Svein Friis, Pål Zeiner

**Affiliations:** 1 Norwegian Institute of Public Health, Marcus Thranes gate 6, 0473 Oslo, Norway; 2 University of Oslo, Oslo, Norway; 3 Oslo University Hospital, Oslo, Norway

**Keywords:** ADHD, ASD, Language difficulties, Behaviour problems, Early childhood education and care, Psycho-social intervention, Special education, Inattention, Hyperactivity/impulsivity, Oppositional behaviour, Mood, Anxiety, Communication, Directed Acyclic Graph, Hierarchical Bayesian modelling

## Abstract

**Background:**

Three to seven percent of pre-schoolers have developmental problems or child psychiatric disorders. Randomized controlled trials (RCTs) indicate that interventions in early childhood education and care (ECEC) improve long-term outcomes of children from disadvantaged backgrounds. It is unknown if such effects generalize beyond the well-structured context of RCTs and to children who may not have a disadvantaged background but have developmental problems or psychiatric disorders.

**Methods:**

We used data from the population-based Norwegian Mother, Father and Child Cohort Study, recruiting pregnant women from 1999 to 2009, with child follow-up from ages 6, 18, and 36 months to ages 5, 7, and 8 years. This sub-study included 2499 children with developmental problems or psychiatric disorders at age five. We investigated the effects of special educational assistance at age five on mother-reported internalizing, externalizing, and communication problems at age eight. We analysed bias due to treatment by indication with directed acyclic graphs, adjusted for treatment predictors to reduce bias, and estimated effects in different patient groups and outcome domains with a hierarchical Bayesian model.

**Results:**

In the adjusted analysis, pre-schoolers who received special educational assistance had on average by 0.1 (0.04–0.16) standardised mean deviation fewer psycho-social difficulties in elementary school.

**Conclusion:**

In a sample of children from mostly higher socioeconomic backgrounds we estimate a positive effects of special educational assistance during the transition from preschool to the school years. It may therefore be considered as an intervention for pre-schoolers with developmental or behaviour problems. More research with improved measurements of treatment and outcomes is needed to solidify the findings and identify success factors for the implementation of special educational assistance in ECEC.

**Supplementary Information:**

The online version contains supplementary material available at 10.1186/s13034-022-00442-5.

## Introduction

Between three and seven percent of pre-schoolers have developmental problems or child psychiatric disorders [1, 2], which are an important risk factor for mental disorders in adulthood [3]. Efforts to promote healthy growth and development in children who struggle in the early years can accordingly improve children’s long-term life opportunities [4]. Indeed, a recent review reported overwhelmingly positive effects of non-cognitive skills on academic, psycho-social, cognitive and health outcomes [5], though effect sizes are typically not large. It has been hypothesized that the effect of interventions decreases as children grow older and therefore, investing resources later, at the age of school entry or beyond, may show less of an effect [6, but also see 7].

Interventions in early childhood are often described as an effective method to improve the long-term outcomes of children from disadvantaged backgrounds [8], or those with specific developmental or behavioural problems like attention deficit hyperactivity disorder, autism, or behaviour or language problems [9]. Interventions in early childhood education and care (ECEC) can be especially effective because, in contrast to parental training programs, their implementation relies less on parents’ abilities or motivation, and on average 93% of three to five year old children in Organisation for Economic Co-operation and Development (OECD) countries are enrolled in ECEC [10, more than 95% or 5 year old children in Norway are in ECEC]. Randomized controlled trials (RCTs) reported clear effects of early interventions in ECEC for a horizon of up to 9 months, for instance for language problems [11], children with ADHD or autism [12], and for teacher classroom management programs [13].

However, the effect sizes of such interventions are not generally large, and less is known about their effect when interventions are provided outside the well-structured context of RCTs. Even though RCTs are, due to their interval validity, the gold standard for estimating treatment effects, differences between study sample and target population and differences in treatment-implementation between study and regular care contexts make a generalization of findings from RCT samples to populations of interest difficult [14–17]. Since RCTs often take place in a controlled setting, it may be difficult to replicate the results in other, less rigid settings. For instance, field professionals in ECEC institutions will draw on a much wider range of sources than formal experimental evidence in order to inform their actions. Thus, while evidence from RCTs is encouraging, it remains unclear how it generalizes to interventions in ECEC provided in regular care.

Only a handful of studies examined the effects of special educational assistance (SEA) interventions in ECEC when they are implemented outside of RCTs. These studies used propensity scores to deal with the problematic internal validity in observational studies—due to treatment by indication—and found that children who received SEA in ECEC showed the same or worse outcomes compared children who did not receive SEA [18, 19].

The Norwegian ECEC-system facilitates the investigation of SEA, because children who cannot fully benefit from standard education and care have the right to receive free SEA. Similar to other OECD countries [2], around 4.5% of pre-schoolers in Norwegian ECEC have impaired functioning. The most common impairment being language and communication difficulties, followed by psycho-social difficulties [20], i.e., the inability to partake in daily activities in a manner that is beneficial to oneself and others due to impaired social or psychological functions. Around 2.6% of pre-schoolers in Norwegian ECEC receive SEA, which is provided for several hours per week and targeted at individual children. Children with language problems typically receive one to three hours SEA a week, and children with combined or more severe developmental problems typically receive more hours SEA (see Additional file 1: Fig. S4). After stimulation of language development, social- and behaviour-training and training of independence are the most frequent types of SEA provided.

In Norwegian ECEC, SEA is provided by individuals with varying qualifications, including personnel with a special education degree, kindergarten teachers, or assistants without specialized training [20]. Most parents (75%) of children with SEA report that ECEC institutions implement SEA with training/pedagogy and social inclusion as equally important goals. Consistently, SEA is typically provided in the context of joint activities of all children and less frequently in one-on-one sessions where child and teacher are isolated from the other children. 80% of parents report that there exists an individualized learning plan for children with SEA, though only 60% report that SEA is implemented according to the plan [20]. To date, no study has–to the best of our knowledge–examined the effect of SEA in ECEC on children’s psycho-social difficulties. Related studies on SEA in Norwegian schools reported that students who received SEA have similar or slightly worse scholastic outcomes compared to those who did not receive it [21, 22, see also 23].

In sum, the few studies examining effects of SEA in ECEC outside the context of RCTs reported small negative, to no effects of SEA. Moreover, most studies focused on educational outcomes, such that the effect of SEA on the development of psycho-social difficulties remains largely unclear. Hence, this large-scale prospective cohort study adds to the existing literature by investigating how SEA in ECEC provided outside RCTs affects the psycho-social development of children with developmental or behavioural problems.

## Methods

### Participants

**Fig. 1:**
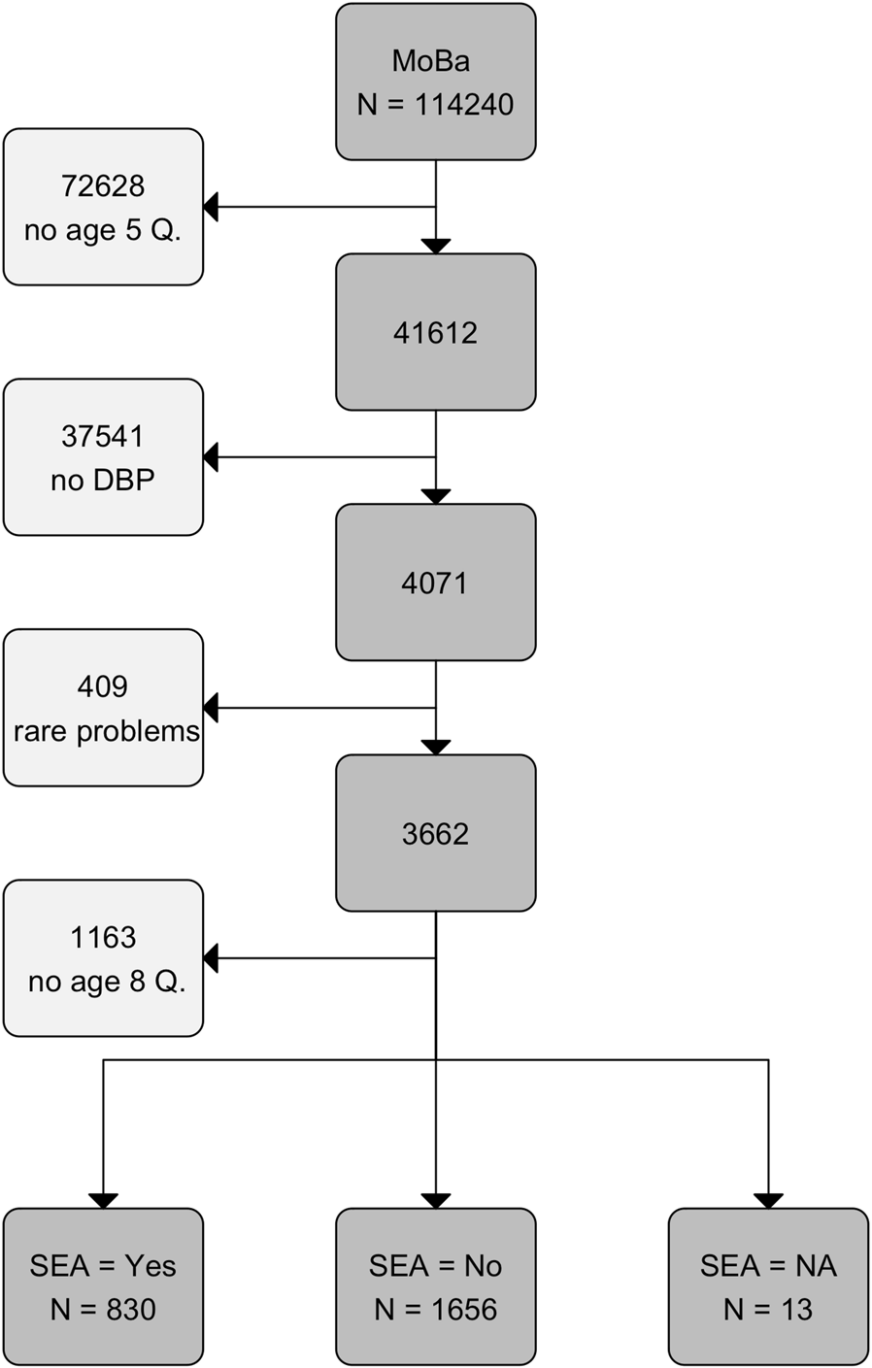
Inclusion flow chart. Age 5 and 8 Q. are MoBa questionnaires sent out at child age five and eight years. Children in the study sample have a parent-reported developmental or behavior problem (DBP) at age five. Children with epilepsy, cerebral palsy, chromosomal defects, severe developmental delay, or hearing loss were excluded from this study. SEA: special educational assistance in early childhood education and care (ECEC)

The study sample is a sub-sample of the Norwegian Mother, Father and Child Cohort Study (MoBa), a prospective population-based pregnancy cohort study conducted by the Norwegian Institute of Public Health [24, 25]. Participating mothers from all over Norway were recruited during routine ultrasound assessment in week 17 or 18 of their pregnancy in the period from 1999 to 2009. 41% of the invited women consented to participate. MoBa participants received questionnaires in gestational week 17 or 18, week 22 and week 30, at child’s age 6 and 18 months, 3, 5, and 8 years and onward. The study is still on-going. The reported analyses also use information from the Medical Birth Registry of Norway [26]. Figure 1 shows the inclusion-flowchart.

The study sample is comprised of children whose mothers indicated in MoBa’s age five year questionnaire that their child had developmental or behaviour problems that were confirmed by a professional or that their child had developmental problems and received special educational support. In addition, we limited the sample to children for whom information about outcomes in the age eight years questionnaire are available (and used statistical methods to deal with loss to follow up). This study focuses on children with one or more of the following developmental or behavioural problems: Attention deficit hyperactivity disorder, language development, oppositional defiant or conduct disorder, autism spectrum disorder and learning disabilities.

### Materials

The current study used rating scales from MoBa questionnaires sent out at child ages five and eight years. Measurements of exposure and inclusion criteria were taken from responses to the five year questionnaire, whereas outcome measures were taken from the eight year questionnaire. The first, 1.5 and three year MoBa questionnaires and the Medical Birth Registry of Norway provided covariates.

#### Exposure

To measure the provision of SEA, we relied on following question: “Is your child receiving now or has he/she received extra help in kindergarten or has he/she been allocated additional resources?” If mothers responded “Yes” to this question, they were additionally asked about the number of hours per week. SEA is provided to individual children, both inside and outside the context of regular preschool activities (see also the introduction section).

#### Outcome variables

**Fig. 2:**
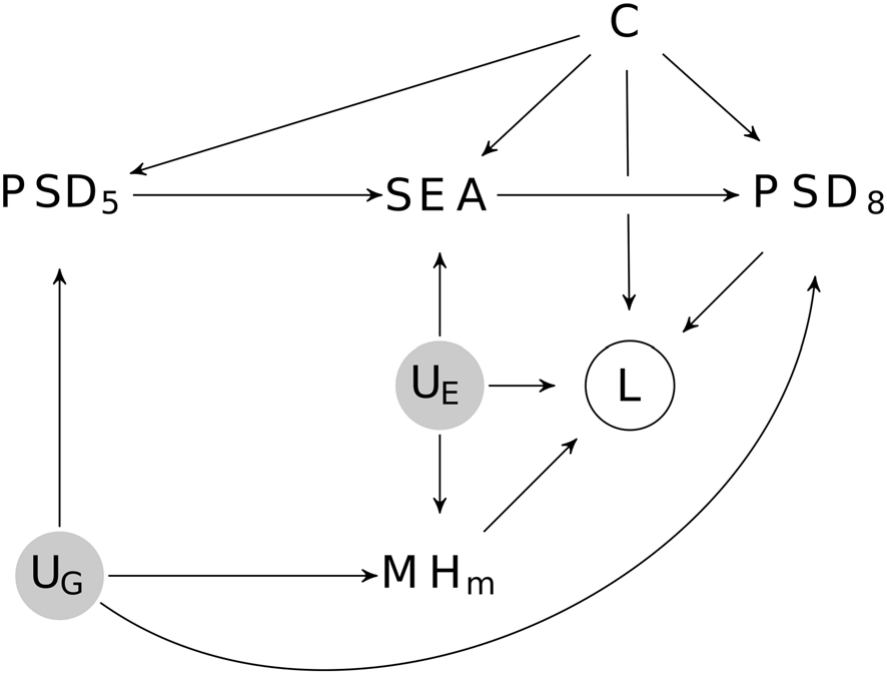
Directed Acyclic Graph of the hypothesized causal relationships between special educational assistance (*SEA*) psycho-social difficulties (*PSD*_*5*_,*PSD*_*8*_), loss to follow up (*L*), maternal mental health (*MH*_*m*_), unobserved environmental and genetic causes (*U*_*E*_,*U*_*G*_) and additional confounders (*C*, i.e. contact with mental health services, maternal education, birth order, birth month, preterm birth). Current and prior psycho-social difficulties *PSD*_*5*_ are confounders causing bias due to treatment by indication and can be controlled through adjustment. Because maternal mental health (*MH*_*m*_) predicts loss to follow up (*L*), which is a collider on a backdoor path between *SEA* and *PSD*_*8*_, loss to follow up has to be controlled through inverse probability weighting

Outcome variables (*PSD*_8_ in Fig. 2) are *sum scores* from various scales about difficulties in psychological and social functioning. Outcome dimensions were attentional, hyperactivity/impulsivity, and behavioural (ODD or CD) problems measured with the Parent Rating Scale for Disruptive Behaviour Disorders (RS-DBD [27]), emotional problems measured with the Short Mood and Feelings Questionnaire (SMFQ [28]) and the Screen for Child Anxiety Related Disorders (SCARED [29]) and communication problems measured with the Children’s Communication Checklist-2 (CCC-2 [30]).

#### Adjustment variables

Adjustment variables and those used to correct for loss to follow up were chosen based on the directed acyclic graph (DAG) shown in Fig. 2. One important set of confounders includes children’s psycho-social difficulties at baseline, because these are causes of treatment and are related to later psycho-social difficulties. A number of scales in MoBa assessed psycho-social difficulties at age five and served as baseline measures (*PSD*_5_ in Fig. 2). These included the Conner’s Parent Rating Scale-Revised, Short Form (CPRS-R (S) [31]), the Child Behavior Checklist (CBCL [32]), the Ages and Stages Questionnaire (ASQ [33]), and the Children’s Communication Checklist-2. While the baseline assessment considers the same mental health and developmental difficulties as the outcome, MoBa used different scales for five and age year old children.

Additional variables used for adjustment or prediction of loss to follow-up included maternal age, education, maternal ADHD symptoms measured with the Adult ADHD Self-Report Scale [34] at child age three and depressive symptoms measured with the SCL-5 [35] at child age five, parity, preterm birth, birth-month, hours special education per week, number of developmental of behaviour problems, and contact with either rehabilitation services, Child and Adolescent Psychiatric Units, or Educational and Psychological Counselling Service at child age 5 years.

### Classification into groups with different developmental or behavioural problems

To classify if and in which area a child had developmental or behavioural problem (DBP), we used MoBa questions about mental health problems at age five. Mothers were asked if their child “suffered, or is currently suffering from any of the following long-term illnesses or health problems.” In addition, mothers were asked if they had been in contact with a Child and Adolescent Psychiatric Unit or the Educational Psychology Counselling Services and if the health problem was confirmed by a professional. Children for whom mothers reported a health problem *and* who indicated that the problem was evaluated by a mental health professional were included in the sample, as were children who had received SEA and for whom mothers reported a health problem.

Disorders or health problems for which MoBa’s age five years questionnaire has questions included Epilepsy, Cerebral Palsy, impaired hearing, which were excluded from the current analysis, together with children for whom mothers indicated a chromosomal defect. MoBa also asked mothers about autism spectrum disorders (ASD), hyperactivity and attention problems (ADHD), language difficulties (Lang), and behavioural problems (Beh). Additional questions about learning disabilities (LD) were also used to identify cases of interest for this study. Each child was classified in one of the following DBP groups: 1. ASD, 2. LD, 3. ADHD & Beh & Lang, 4. ADHD & Beh, 5. ADHD & Lang, 6. ADHD, 7. Lang, 8. Beh. For some children, mothers indicated multiple DBP, in which case the child was assigned to the first group it fell into. If, for example, a mother indicated ASD, ADHD and language problems, the child was assigned to the ASD group (details in Additional file 1 and Table S1). The rational underlying this classification scheme was to use existing psychiatric diagnoses, and to classify children according to their most impairing problem because these have typically stronger and more persistent associations with psycho-social development.

### Data analysis

All analyses were performed using R [36]. The Bayesian hierarchical regression model was estimated with the brms package [37]. The analyses are described in more detail in the Additional file 1.

#### Bias from treatment by indication and loss to follow up

Estimation of treatment effects from observational data is difficult because treatment is not assigned randomly. Instead, individuals with more psycho-social difficulties at age five, who are also more likely to have psycho-social difficulties in the future, more likely receive treatment (treatment by indication). In addition, loss to follow up makes estimation of treatment effects difficult. Therefore, we used a directed acyclic graph [DAG, 38, see Fig. 2] to explicate the assumed causal structure and to determine with which approach to deal with potential biases. Given this structural model, inverse probability of continued participation weighting was needed to reduce bias from loss to follow up [39], whereas adjustment for common causes of SEA and psycho-social difficulties at age eight was sufficient to control bias from treatment by indication. This means that we effectively estimated the effect of SEA on the change of psycho-social difficulties from preschool to elementary school.

#### Estimation of the treatment effects

We used a Bayesian adjusted and weighted hierarchical ordinal regression to estimate effects of SEA [37, 40, 41]. A hierarchical regression induces partial pooling (shrinkage) of estimates, which reduces the variance of estimates [42] and controls the multiple comparison problem [43]. Importantly, when analysing related patient groups, hierarchical regression results in more accurate association estimates than independent analysis of these groups [42]. We used an ordinal regression model, because the estimation of latent, normally distributed traits that underlie the rating-scale responses facilitates the presentation of results in terms of standardized mean differences (SMD). To deal with missing data, reported results were obtained by pooling over the independent analyses of the 50 imputed data sets [44]. Consistent with recent recommendations to focus on estimation of effect sizes instead of significance testing [45, 46] we generally report mean effect sizes and the 90% credible intervals.

## Results

The study sample includes 2499 participants (c.f., Fig. 1). Thirty-three percent of the children in the sample received SEA. Table 1 describes the study sample. Additional file 1: Figs. S4 and S5 show that children with more severe problems (e.g. ASD) were more likely to receive SEA and also received SEA from better educated personnel.

**Table 1:** Study sample

Variable	W/o SEA	With SEA	Total
Special educational assistance (SEA)			
Boy	1063 (63.8%)	586 (70.3%)	1649 (66%)
Girl	602 (36.2%)	248 (29.7%)	850 (34%)
Hours	0 (0, 0)	4.76 (1, 6)	1.59 (0, 1)
Developmental or behaviour problem (DBP) group			
ASD	11 (0.7%)	32 (3.8%)	43 (1.7%)
LD	19 (1.1%)	63 (7.6%)	82 (3.3%)
ADHD & Beh & Lang	12 (0.7%)	19 (2.3%)	31 (1.2%)
ADHD & Lang	58 (3.5%)	85 (10.2%)	143 (5.7%)
ADHD & Beh	108 (6.5%)	38 (4.6%)	146 (5.8%)
ADHD	330 (19.8%)	71 (8.5%)	401 (16%)
Lang	847 (50.9%)	486 (58.3%)	1333 (53.3%)
Beh	280 (16.8%)	40 (4.8%)	320 (12.8%)
Psycho-social difficulties (PSD) at child age five			
Attention	6.03 (2, 9)	6.98 (2, 10)	6.34 (2, 9)
Hyperactivity	4.67 (3, 6)	4.68 (3, 6)	4.67 (3, 6)
Externalizing (CBCL)	3.98 (2, 6)	3.73 (1, 6)	3.9 (2, 6)
Internalizing (CBCL)	2.01 (0, 3)	2.16 (0, 3)	2.06 (0, 3)
Communication (CCC)	3.93 (2, 6)	4.76 (3, 7)	4.21 (2, 6)
Development (ASQ)	1.34 (0, 2)	2.31 (1, 3)	1.67 (0, 2)
Psycho-social difficulties (PSD) at child age eight			
Attention (ATT, RS-DBD)	7.51 (4, 10)	8.3 (4, 12)	7.77 (4, 11)
Hyperactivity (HYP, RS-DBD)	6.07 (2, 9)	5.77 (1, 8)	5.97 (2, 9)
Oppositional (OPP, RS-DBD)	5.18 (2, 7)	4.44 (1, 6)	4.93 (2, 7)
Mood (MOOD, SMFQ)	3.06 (1, 4)	2.96 (1, 4.75)	3.03 (1, 4)
Anxiety (ANX, SCARED)	1.21 (0, 2)	1.22 (0, 2)	1.21 (0, 2)
Communication (COMM, CCC)	7.75 (4, 11)	10.29 (5, 14)	8.6 (4, 12)
Maternal characteristics			
Education (years)	14.01 (12, 15)	13.98 (12, 16)	14 (12, 15)
Age (years)	30.52 (27, 34)	30.82 (28, 34)	30.62 (28, 34)
ADHD (ADHD-RS)	7.38 (5, 10)	7.15 (5, 9)	7.3 (5, 10)
Depression (SCL-5)	2.53 (0, 4)	2.43 (0, 3)	2.5 (0, 3)

ASD: Autism spectrum disorder; LD: Learning difficulties; Lang: Language problems; Beh: behaviour problems; SEA: special educational assistance. Abbreviations and original scales for PSD are given in parentheses (see Methods section for full names). Numbers in parentheses are percent or first and third quartile

Inverse probability weights reduced the differences in mean values for covariates between participants followed up and those lost to follow up to less than 0.1 SMD (c.f. Additional file 1: Fig. S1; [47]). Cumulative distribution plots showed that weighting balanced the entire distributions of covariates (Additional file 1: Figs. S7 and S8).

### Effects of special educational assistance

Consistent with the structural model shown in Fig. 2, the analysis without adjustment showed that SEA at age five was associated with more psycho-social difficulties at age eight (c.f. Additional file 1: Table S3 and Fig. S7). Additional file 1: Table S4 and Figs. S9 and S10, S11, and S12 show coefficients of the adjusted regression model, which indicates that after adjustment for confounders SEA was associated with less psycho-social difficulties at age eight.

**Fig. 3:**
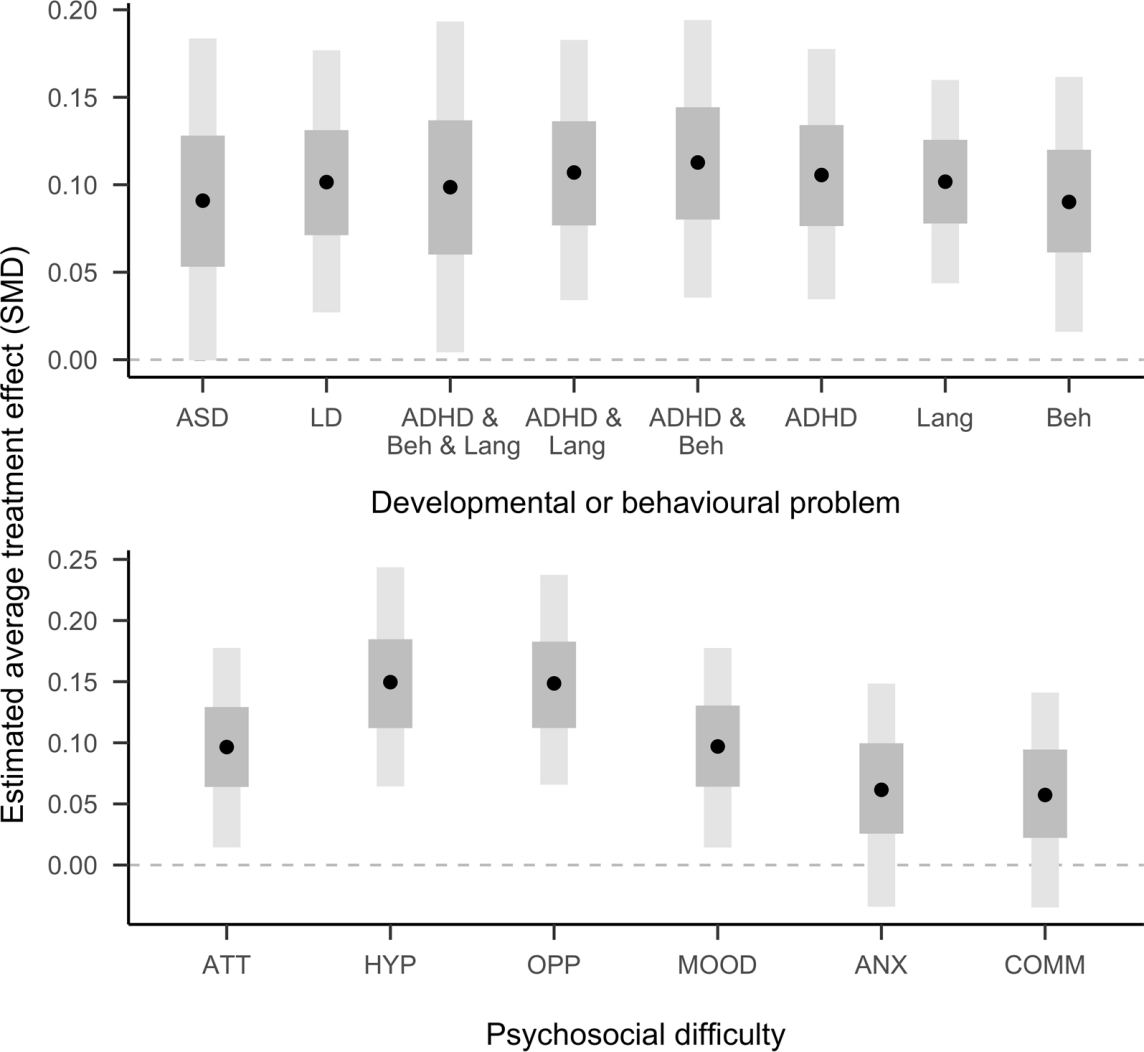
Estimated average treatment effects stratified by group (top) and outcome (bottom). Points indicate means, grey and dark-grey bands indicate 50% and 90% credible intervals. SMD: standardized mean deviation. Abbreviations as in Table 1

Over all psycho-social outcomes and groups of developmental or behaviour problems the estimated average treatment effect (ATE) was a symptom reduction by 0.10 standardized mean deviations (SMD) (Credible Interval CI 0.04, 0.16). Figure 3 shows that the 90% credible interval is for all groups above 0. The pairwise comparisons of all groups did not show clear differences in the estimated treatment effects between groups (c.f. Additional file 1: Table S5 and Fig. S14). Figure 3 and Table 2 also show estimated effect sizes stratified by outcomes and indicate that SEA had a positive effect on all measured psycho-social outcomes. While there were some differences in the effect size estimates for different outcomes, in particular smaller effects for anxiety and communication problems, pairwise comparisons did not show reliable differences between them (c.f. Additional file 1: Table S6 and Fig. S15). Effect size estimates did not vary substantially by the child sex (c.f. Additional file 1: Fig. S18).

**Table 2:** Estimated average treatment effects (ATE) stratified by groups with different developmental and behavioural problems (rows) and psycho-social difficulties (columns)

Group	ATT	HYP	OPP	MOOD	ANX	COMM	Average
ASD	0.08 (− 0.06, 0.21)	0.11 (− 0.02, 0.25)	0.11 (− 0.02, 0.25)	0.1 (− 0.03, 0.24)	0.08 (− 0.06, 0.21)	0.07 (− 0.08, 0.2)	0.09 (0, 0.18)
LD	0.09 (− 0.02, 0.21)	0.11 (0, 0.23)	0.11 (0, 0.23)	0.11 (0, 0.23)	0.1 (− 0.01, 0.22)	0.08 (− 0.05, 0.19)	0.1 (0.03, 0.18)
ADHD & Beh & Lang	0.1 (− 0.04,0.24)	0.11 (− 0.03,0.26)	0.09 (− 0.05, 0.23)	0.11 (− 0.03, 0.25)	0.1 (− 0.04, 0.25)	0.08 (− 0.07, 0.22)	0.1 (0, 0.19)
ADHD & Lang	0.07 (− 0.05, 0.18)	0.11 (− 0.01,0.22)	0.13 (0.02, 0.26)	0.1 (− 0.01, 0.21)	0.13 (0.02, 0.26)	0.1 (− 0.02, 0.21)	0.11 (0.03, 0.18)
ADHD & Beh	0.15 (0.03, 0.29)	0.11 (− 0.01,0.23)	0.11 (− 0.01, 0.24)	0.1 (− 0.02, 0.22)	0.11 (− 0.01, 0.23)	0.09 (− 0.03, 0.22)	0.11 (0.04, 0.19)
ADHD	0.09 (− 0.02, 0.2)	0.12 (0.02,0.23)	0.14 (0.03, 0.26)	0.09 (− 0.03, 0.19)	0.11 (0, 0.23)	0.08 (− 0.03, 0.19)	0.11 (0.03, 0.18)
Lang	0.1 (0.01, 0.18)	0.15 (0.06,0.24)	0.15 (0.07, 0.24)	0.1 (0.01, 0.18)	0.06 (− 0.03, 0.15)	0.06 (− 0.03, 0.14)	0.1 (0.04, 0.16)
Beh	0.09 (− 0.03, 0.2)	0.07 (− 0.06, 0.18)	0.11 (0, 0.24)	0.06 (− 0.07, 0.17)	0.11 (0, 0.23)	0.1 (− 0.02, 0.21)	0.09 (0.02, 0.16)
Average	0.1 (0.01, 0.18)	0.15 (0.06, 0.24)	0.15 (0.07, 0.24)	0.1 (0.01, 0.18)	0.06 (− 0.03, 0.15)	0.06 (− 0.03, 0.14)	

ATEs are reported as standardised mean differences (SMD). Numbers are means (90% credible intervals)

## Discussion

This research used observational data from a longitudinal population based prospective cohort study to investigate the effect of special educational assistance (SEA) in ECEC on psycho-social difficulties of children with developmental or behaviour problems. We found that, after adjustment for treatment indicators, mothers of children who received SEA in kindergarten reported fewer psycho-social difficulties three years later, compared to mothers whose children did not receive SEA.

While there was some variation in the extent of the positive effect of SEA between groups and different psycho-social difficulties, these differences were not reliably different from zero (c.f. Additional file 1: Figs. S14 and S15). Because the credible intervals for these differences are large compared to the magnitude of the estimated overall effect and the random effects standard deviations are clearly non-zero (S4), these results do not exclude the possibility of group differences. Instead, they might reflect difficulties in reliably measuring exposure, covariates, and outcomes based on parent reports only. Still, the available data were sufficient to reveal an overall positive effect of SEA.

While the positive effect reported in this study is consistent with the results of randomized controlled trials [12, 13] and with reports of the positive effects of preschool child care quality [48], it also stands in contrast to previous observational studies, which estimated no or a small negative “effects” of special education. This apparent contradiction can be due to a number of differences between the current and previous studies. We had estimates of pre-treatment difficulties, and could estimate effects of special education on the change of psycho-social difficulties. Moreover, we used adjustment for treatment predictors instead of propensity score weighting. Adjustment is the preferable approach if treatment-predictors are not colliders on a backdoor path from the outcome to the treatment and if the sample size is large enough to allow for inclusion of many adjustment variables. Another important difference is that whereas previous studies focused on scholastic outcomes, we focused on the effect on psycho-social difficulties. This is a to date little examined but important outcome of SEA, because early psycho-social difficulties are associated with impaired functioning in adulthood [3]. Interestingly, the clear results of SEA on externalizing behaviour suggests that, in addition to helping children with DBP, it can also benefit their families by reducing disruptive behaviour.

The estimated effect size for the reduction of psycho-social difficulties is with on average 0.10 standardized mean difference small. In comparison, previous meta analysis about school– or ECEC–based interventions found effect sizes of between -0.3 and 1.3 SMD for children with or at risk for ADHD [49, 50] or SMDs between 0.3 and 1.1 for children with autism [51]. Randomized trials of classroom management training for kindergarten teachers showed effect sizes similar to our results [Cohen’s d around 0.3 for high risk children at the nine-months follow up, 52]. A recent meta-analysis of reported effect sizes around 0.2–0.3 SMD from experimental manipulations of non-cognitive skill on psycho-social outcomes [5], and smaller effects around 0.1 SMD from non-experimental longitudinal studies. It is possible that the small effect sizes we estimated are, in addition to above mentioned measurement problems, due to the fact the SEA was often provided by personnel with limited training, especially for children with typically less severe problems (c.f. Additional file 1: Fig. S5).

More generally, the decentralized organization of the Educational and Psychological Counselling Service is likely to lead to a large variation in the implementation of SEA [53]. MoBa did not collect more detailed data about SEA, which could help to elucidate when it is most effective. Another possible explanation is that the composition of the study sample, which over-represents well-educated families compared to the population [39], leads to an underestimation of the true effect size, because well-educated parents could reduce children’s psycho-social difficulties even without SEA [54].

While the current study showed that mothers reported fewer psycho-social difficulties in elementary school when their children received SEA in ECEC, a causal interpretation of this result as reflecting an effect of SEA rests on a number of assumptions encoded in Fig. 2. One un-testable assumption is that there are no unmeasured confounders that predict both which children receive SEA and their developmental pathway. Even though the reported analysis includes obvious confounders, other unobserved confounders like e.g. parental engagement could still account for some of the positive association of SEA and psycho-social development. However, because RCTs of SEA and similar interventions typically report positive effects, and thus confirm a causal role of SEA, it appears unlikely that the effects estimated in this study are primarily due to confounding.

The current study has a number of limitations that should be addressed in future studies. Outcomes should be assessed through blinded raters or objective instruments and the quality and quantity of the treatments need to be assessed in greater detail. Moreover, it is important that study samples include participants with a higher a priori prevalence of mental health problems (i.e. no over-representation of highly educated parents which characterizes MoBa) and that care is taken to avoid loss to follow up. Better measurements and more representative samples will be useful to investigate reasons for the relatively small effects observed in the current study, and to identify criteria for effective interventions in ECEC.

## Conclusion

Previous RCTs about special educational assistance and teacher management programs showed that interventions in ECEC have a positive immediate impact for children with developmental or behavioural problems, but provide little guidance on long-term effects. The current study has, due to its observational character, a lower internal validity than RCTs, but complements them in terms of external validity and by examining long-term effects. It thus strengthens the view that interventions in ECEC are a useful approach to support pre-schoolers with developmental or behavioural problems.

In sum, the current study suggests that the psycho-social development of children with developmental or behaviour problems may be modified in a positive way through interventions in ECEC, also when provided outside the structured context of randomized controlled trials. Future research with better measurements and more representative samples should investigate under which conditions such interventions are most effective.

## Supplementary Information

**Additional file 1.** Supplementary methods and results.

## Data Availability

The data-set supporting the conclusions of this article is available upon application to Norwegian Mother, Father and Child Cohort Study (MoBa, https://www.fhi.no/en/studies/moba/.
